# Association between the Planetary Health Diet Index and biological aging among the U.S. population

**DOI:** 10.3389/fpubh.2024.1482959

**Published:** 2024-10-22

**Authors:** Shaoqun Huang, Haoying Hu, Hongyang Gong

**Affiliations:** ^1^Department of Oncology Surgery, Fuzhou Hospital of Traditional Chinese Medicine Affiliated to Fujian University of Traditional Chinese Medicine, Fuzhou, Fujian, China; ^2^College of Clinical Medicine, Chengdu University of Traditional Chinese Medicine, Chengdu, Sichuan, China; ^3^Department of Gastroenterology, China Academy of Chinese Medical Sciences, Wangjing Hospital, Beijing, China; ^4^Department of Physiology, College of Medicine, Chosun University, Gwangju, Republic of Korea

**Keywords:** Planetary Health Diet Index, biological aging, NHANES, U.S. population, association

## Abstract

**Background:**

The Planetary Health Diet (PHD) is a novel dietary pattern proposed by the EAT-Lancet Commission in 2019, yet a limited study has investigated the anti-aging effects of PHD to date.

**Objectives:**

This study aimed to explore the association between adherence to PHD, as quantified by the Planetary Health Diet Index (PHDI), and biological aging in American populations.

**Methods:**

Data were obtained from the National Health and Nutrition Examination Survey (NHANES) for 1999–2018. Food consumption information was relied on two 24-h diet recall questionnaires. The biological aging condition was comprehensively assessed by four biological markers, including phenotypic age, biological age, telomere length, and klotho concentration. Weighted multivariate linear models, restricted cubic spline (RCS), and subgroup analysis were subsequently carried out to evaluate the influence of PHDI on biological aging.

**Results:**

44,925 participants with complete data were finally enrolled in our study. The fully adjusted models showed decreased 0.20 years in phenotypic age [−0.20 (−0.31, −0.10)] and declined 0.54 years in biological age [−0.54 (−0.69, −0.38)] correlated with PHDI per 10 scores increment. Klotho concentration [6.2 (1.0, 11.0)] was positively related to PHDI. In Model 2, telomere length increased by 0.02 bp for every 10-point rise in PHDI. Besides, the RCS analysis results exhibited a curvilinear relationship between PHDI and four indicators.

**Conclusion:**

Our study explored a significant correlation between PHDI and biological aging, indicating that adherence to PHD may prevent biological aging.

## 1 Introduction

Aging is characterized by the gradual degeneration in muti-system physiological and functional abilities (1, 2), as the leading cause of most chronic diseases and mortality (3). According to the report, the amount of population surpassing 60 years old will double to nearly 2.1 billion by 2050 (4, 5). With the growing trend of population aging, biological aging, which involves irreversible changes in the physiochemistry and metabolism of cells (6), has emerged as a global public health concern. Worse still, numerous studies showed that accelerated biological aging has positive correlations with adverse outcomes, such as cancer, rheumatoid arthritis, and some mental disorders (7–9). Since chronological age (CA) cannot precisely reflect varying rates of aging, measures of biological aging identifying individuals who are “aging faster” have been developed, which were mainly based on biomarkers (e.g., telomere length, DNA methylation age, and klotho) and different aging models (e.g., phenotypic age and biological age) (10–12). Generally speaking, biological age denotes chronological age at the same physiological function, whereas phenotypic age relates to chronological age at the same risk of death. Biological age and phenotypic age determined from clinically visible data are thought to be more accurate indicators of the course of aging (13). Today, more than ever before, exploring valuable intervention strategies is essential to prolonging a healthy lifespan and managing the difficulties posed by biological aging.

Diet is one of the most susceptible areas for intervention in effectively modifying the landscape of biological aging, and this fact has been proved by multiple lines of evidence. For instance, a large cohort study in North America reported that intakes of specific food groups, including but not limited to nuts, peaches, and discretionary oil, have positive effects on retarding biological aging acceleration (14). Another study suggested that diets abundant in fruits, vegetables, fish, *etcetera*, can slow down aging clocks and promote healthy aging in different ways (15). However, these studies failed to provide comprehensive and reliable dietary guidance for retarding biological aging owing to the ignorance of the fact that the daily diet is the combination of food groups and nutrients rather than isolation.

Apart from concentrating on simple food groups, multiple studies are shedding light on the ability of complicated dietary patterns to delay the biological aging pathological progression, mainly encompassing the ketogenic diet and Mediterranean diet (MedDiet) (16). Nevertheless, due to the escalating global population, these dietary patterns do not effectively address the double challenges of environmental sustainability and public diet nutrition. In response to this concern, the EAT-Lancet Commission proposed the Planetary Health Diet (PHD) in 2019, encouraging individuals to consume high-quality plant-based food, such as fruits, vegetables, and whole grains (17). Additionally, this diet emphasizes reducing red meat, processed food, and sugar, which mainly contribute to environmental degradation and climate change (18). Unlike other dietary patterns predominantly focused on current health outcomes, the PHD represents a significant advancement in maintaining both individual well-being and long-term planet health, making it a valuable strategy for further research and adoption (19).

Recently, rising evidence demonstrated negative associations between PHD and chronic diseases, including stroke, heart failure, atrial fibrillation, and type 2 diabetes (20–23). Up to now, there is still a need for research to investigate its specific effects on the development of biological aging. Given the high dietary quality of PHD (24), we hypothesized that adherence to the PHD would slow down biological aging and verified it by assessing the relationship between the Planetary Health Diet Index (PHDI) and biological aging by taking advantage of large sample data from the National Health and Nutrition Examination Survey (NHANES).

In the context of global environmental changes and health challenges, assessing the impact of dietary patterns on biological aging is of paramount importance. While previous studies have explored the relationship between diet and health (25–28), systematic investigations into the association between the Planetary Health Diet Index and biological aging remain limited, particularly within the U.S. population. Therefore, our study not only addresses this academic gap but also provides critical insights for public health policy, promoting sustainable dietary practices that enhance healthy aging.

## 2 Methods

### 2.1 Study design and population

The NHANES is intended to evaluate the nutritional and healthy condition of the U.S. population. Data from the NHANES are large-sample, high-quality, and representative to facilitate valuable research on different health conditions in the general population (29). The NHANES study protocol was approved by the NCHS research ethics review board, and participants gave written informed permission at the time of recruitment.^1^ Since this study was based on deidentified data that was made publically available, no ethical approval nor permission was needed.

**Figure 1:**
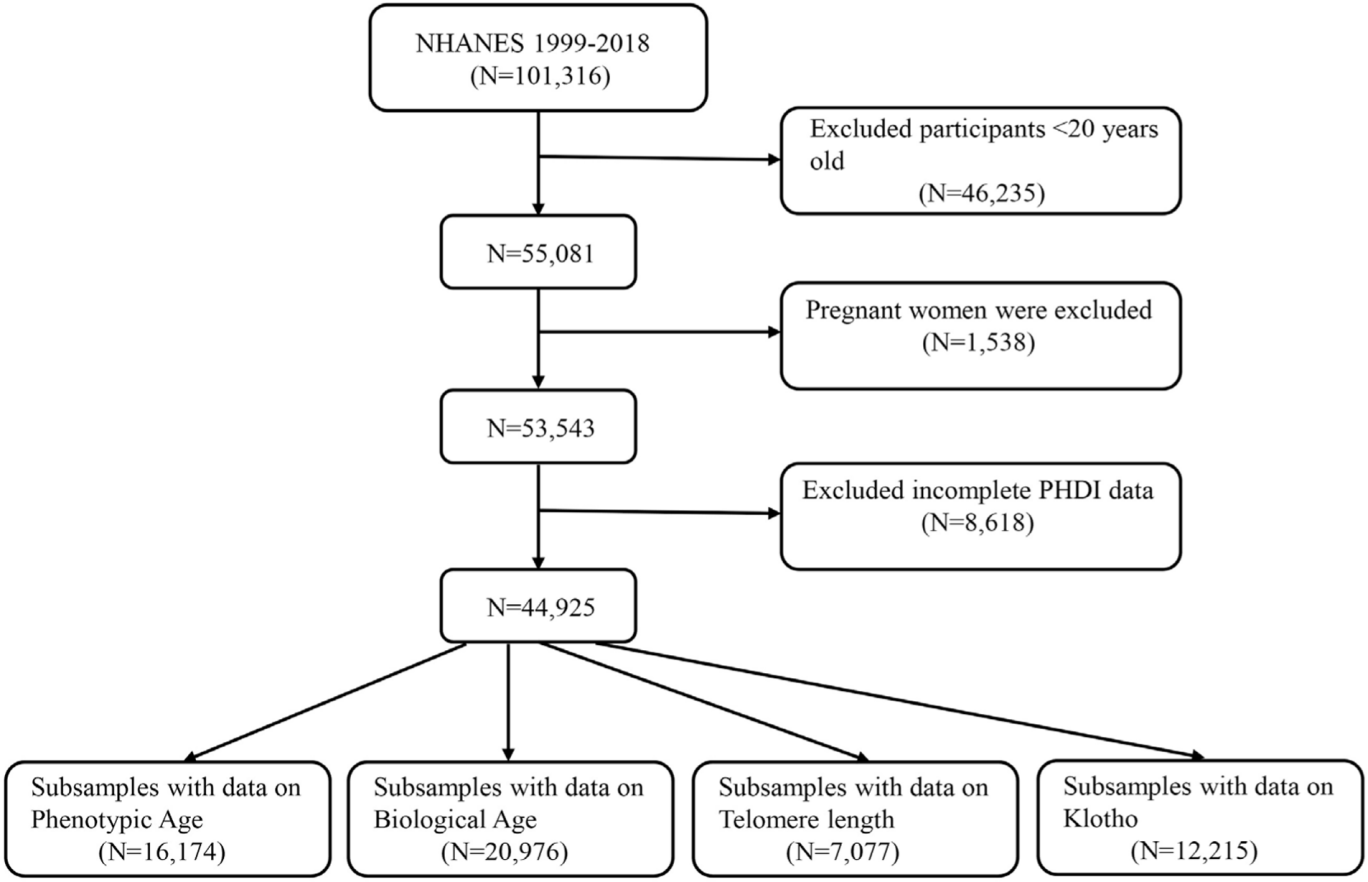
A flow diagram of eligible participant selection in the National Health and Nutrition Examination Survey.

Data were obtained from the NHANES for 1999–2018, comprising nearly 20 years. We first excluded participants under 20 years old and pregnant women and further excluded participants with incomplete PHDI data. Ultimately, 44,925 eligible participants were enrolled in this study. Based on four aging-related indicators, all the participants were classified into four groups: Phenotypic age (*N* = 16,174), Biological age (*N* = 20,976), Telomere length (*N* = 7,077), and Klotho (*N* = 12,215). More details are listed in Figure 1.

### 2.2 Evaluation of PHDI

The PHDI is a relatively new calorie-based index developed by Cacau et al. according to the reference diet mentioned before (30). We incorporated various calculating methods for evaluating PHDI (31, 32). The Planetary Health Diet Index (PHDI) serves as a detailed dietary assessment tool aimed at evaluating how well individuals adhere to the dietary guidelines established by the EAT-Lancet Commission. These guidelines underscore the significance of a diet that supports both human health and environmental sustainability, advocating for a diet rich in plant-based foods while limiting the consumption of animal-derived products. The PHDI is organized into 14 components, each representing different aspects of dietary intake that are in line with the EAT-Lancet recommendations. These components are categorized into two main groups: (1) Adequacy Components: This category focuses on the consumption of foods that are recommended for their health and environmental benefits, such as whole grains, fruits, vegetables, nuts, seeds, legumes, and unsaturated oils. Each of these components is scored on a scale from 0 to 10, with higher scores indicating better adherence to the suggested intake levels. (2) Moderation Components: This group emphasizes reducing the intake of foods that should be consumed sparingly, including red and processed meats, dairy, poultry, eggs, fish, saturated fats, trans fats, added sugars, and fruit juices. Like the adequacy components, these are also scored from 0 to 10, where higher scores represent lower consumption of these foods. The total PHDI score can range from 0 to 140, with higher scores signifying a greater alignment with the EAT-Lancet dietary guidelines. The score for each component is calculated based on the participant’s reported food intake, which is then compared to predefined standards derived from the midpoint of the recommended intake ranges provided by the EAT-Lancet Commission. Supplementary Table S1 provides specific procedures for estimating the PHDI.

### 2.3 Dietary data

Dietary data encompasses detailed information on individuals’ food and beverage intake, playing a vital role in evaluating dietary patterns and nutritional quality. In research, such as studies utilizing the PHDI, dietary data is commonly collected through 24-h dietary recalls. Participants are asked to recall everything they ate and drank during the previous day, offering a snapshot of their eating habits. This information is then processed using tools like the Food Patterns Equivalents Database (FPED), which classifies foods into specific components based on their nutritional content. The FPED converts food items into standardized units, such as grams or cup-equivalents, enabling accurate comparison and analysis. For instance, multi-ingredient dishes are broken down into their ingredients, which are then assigned to the appropriate food categories. In this study, dietary intake data was obtained through two 24-h dietary recall interviews, and the average of the combined data from both days was used. It is important to note that all NHANES participants were eligible for two 24-h dietary recall interviews due to potential biases in personal memory. The first dietary recall was conducted in person at the Mobile Examination Center (MEC), while the second interview took place via phone 3–10 days later. To mitigate recall bias and ensure accuracy, we typically use the average of the two 24-h dietary recalls in our analyses. Specific methods for calculating the PHDI are provided in Supplementary Table S1.

### 2.4 Assessment of biological aging

To comprehensively assess the biological aging condition, we selected four multidimensional indicators that are strongly correlated with it. Phenotypic and biological age are two novelty metrics integrating various clinical parameters to depict the aging process from molecular to organ and system levels (33). In line with previous research, nine biomarkers (e.g., C-reactive protein, albumin, glucose, mean cell volume, alkaline phosphatase, lymphocyte percentage, white blood cell count, creatinine, and chronological age) and eight biomarkers (e.g., systolic blood pressure, serum urea nitrogen, serum total cholesterol, serum creatinine, serum alkaline phosphatase, serum albumin, glycated hemoglobin, and C-reactive protein) were included in calculating phenotypic age and biological age, respectively (12, 34). The algorithm for phenotypic age was based on the accepted method published by Levine et al., and biological age was conducted utilizing the most accurate calculating method from Klemera et al. (35, 36). Detailed information about the calculation can be found in Supplementary material. To measure relative leukocyte telomere length, the quantitative polymerase chain reaction (qPCR) method was used. This method calculates the proportion between telomere repeat copies number and single copy gene copies number for reference and DNA samples (Specific formulae are detailed in the Supplementary material). As for evaluating serum klotho levels, IBL International, Japan, provided commercially accessible ELISA kits to analyze serum samples persevered at−80°C, with a 6 pg/mL sensitivity. After analyzing each sample repeatedly, the final result of the sample was calculated as the mean of the 2 concentrations. Complete descriptions of telomere length and klotho evaluation were provided in the Cawthon RM et al. study and Zhang Z et al. study, respectively (37, 38). Additionally, the laboratory assessments of telomere length and serum Klotho levels may have some potential limitations. However, within the NHANES database, each blood sample for telomere length measurement was tested three times on three different days, with outliers excluded from the analysis. For serum Klotho concentration, each sample was analyzed twice, and the results were averaged to ensure accuracy.

### 2.5 Covariates

Covariates considered as potential confounders were incorporated in this study to mitigate potential biases. The Supplementary Table S2 contains a detailed classification of covariates.

### 2.6 Statistical analysis

The current study applied the NCHS-suggested weights to ensure that all data was nationally representative. The new weights (for 2005–2018) were calculated as 1/7 × the two-day dietary sample weight (WTDR2D), which served as the weighting variable. The *t*-tests [expressed as mean (standard deviation, SD)] were used for continuous (per 10 score increase) variables, and chi-square tests (expressed as percentages) were utilized for categorical variables. We divided PHDI into Q1-Q4 groups according to quartiles while regarding it as a categorical variable. The analysis involved weighted multivariate linear regression models investigating the relationship between PHDI and four biological aging indicators. The outcomes were displayed as a *β* with a 95% confidence interval (CI). Three covariate-adjusted models were evaluated: Model 1 had no covariates, Model 2 primarily adjusted for demographic characteristics, and Model 3 adjusted for all variables. After that, to determine whether PHDI and four biological aging indicators had dose–response relationships, we employed the restricted cubic spline (RCS) during this evaluation. Potential interactions were subsequently examined via multiplicative interaction analysis based on prespecified stratified terms. No previous statistical power computation was done because the sample size was established using the available data. All analyses were performed by the R (v.4.3.1) statistical software, and two-tailed *p* values below 0.05 were taken as statistically significant.

## 3 Results

### 3.1 Baseline characteristics of participants

We stratified 44,925 participants depending on PHDI scores, and their average age was 48.01 years, with more than half (51.61%) female population (Table 1). On average, the phenotypic age of overall participants was 31.12 years, while 31.55 years for biological age, 5.81 for telomere length, and 842.74 pg/mL for klotho. Compared with individuals in the Q1 group, the Q4 group was older, with more non-Hispanic whites, higher educational levels, better income conditions, and more female population. Additionally, individuals in the lowest PHDI quartile demonstrated having habits of smoking and heavy drinking.

**Table 1:** Baseline characteristics of all participants were stratified by PHDI quartile.

Characteristic	Overall	Planetary Health Diet Index				*p*-value
		Q1	Q2	Q3	Q4	
Age (years) [Mean(SD)]	48.01 (17.10)	43.91 (16.26)	47.51 (17.08)	49.54 (17.19)	51.08 (17.00)	<0.001
Gender (%)						<0.001
Male	48.39	56.57	50.99	46.61	39.40	
Female	51.61	43.43	49.01	53.39	60.60	
Race (%)						<0.001
Non-Hispanic White	69.99	67.60	69.55	71.35	71.46	
Non-Hispanic Black	11.04	14.14	11.50	9.67	8.87	
Mexican American	7.33	8.06	7.98	7.09	6.18	
Other	11.64	10.19	10.98	11.89	13.49	
Married/live with partner (%)						<0.001
No	36.73	39.69	37.35	34.96	34.90	
Yes	63.27	60.31	62.65	65.04	65.10	
Education level (%)						<0.001
Below high school	16.79	18.74	18.27	16.67	13.47	
High School or above	83.21	81.26	81.73	83.33	86.53	
PIR (%)						<0.001
Not Poor	78.96	75.05	77.18	80.08	83.58	
poor	21.04	24.95	22.82	19.92	16.42	
Smoking (%)						<0.001
Never	53.28	48.36	50.57	54.44	59.77	
Former	25.53	22.48	25.59	26.76	27.31	
Current	21.18	29.16	23.84	18.80	12.92	
Drinking (%)						<0.001
Former	14.80	14.23	14.57	15.71	14.68	
Heavy	20.50	28.29	22.70	18.04	12.99	
Mild	36.23	31.27	33.62	37.09	42.93	
Moderate	16.95	17.56	17.19	16.92	16.13	
Never	11.52	8.65	11.92	12.24	13.28	
Hypertension (%)						0.025
No	61.76	63.40	60.88	61.11	61.66	
Yes	38.24	36.60	39.12	38.89	38.34	
Diabetes (%)						0.087
No	86.87	87.08	86.90	86.04	87.46	
Yes	13.13	12.92	13.10	13.96	12.54	
High cholesterol (%)						<0.001
No	61.85	65.46	61.64	60.21	60.26	
Yes	38.15	34.54	38.36	39.79	39.74	
Phenotypic Age (years) [Mean (SD)]	31.12 (9.79)	31.00 (9.85)	31.31 (10.03)	31.15 (9.73)	31.03 (9.50)	0.734
Biological Age (years) [Mean (SD)]	31.55 (12.53)	32.07 (12.99)	31.92 (12.75)	31.40 (12.15)	30.58 (11.95)	<0.001
Telomere length [Mean (SD)]	5.81 (0.64)	5.87 (0.63)	5.78 (0.64)	5.81 (0.63)	5.81 (0.65)	0.009
Klotho (pg/mL) [Mean (SD)]	842.74 (293.76)	829.63 (297.42)	828.44 (291.90)	850.32 (282.44)	863.08 (301.52)	<0.001

Mean (SD) for continuous variables. Percentages (weighted *N*, %) for categorical variables. The *p*-value was calculated using a chi-square test and Students *T* test after considering the sampling weights.

PIR, Ratio of family income to poverty; PHDI, Planetary Health Diet Index.

### 3.2 Association between PHDI and biological aging

Weighted multivariate linear regression results of all unadjusted and adjusted models are listed in Table 2. There was a significantly inverse association between PHDI and phenotypic age, and the results remained the same even in the fully adjusted model, which demonstrated decreased 0.20 years in phenotypic age with PHDI per 10 scores increment [−0.20 (−0.31, −0.10)]. It was suggested that a similar relationship pattern existed between PHDI and biological age, with a 0.54-year decline [−0.54 (−0.69, −0.38)] in biological age connected with a 10-score increase in PHDI based on model 3. After categorizing PHDI into four groups, the above linear correlations notably persisted among phenotypic age and biological age intervals driven by trend tests. Compared with the bottom group participants, those in the top PHDI group experienced a 0.82-year decline in phenotypic age and a 1.80-year decline in biological age, as indicated below.

**Table 2:** Associations between PHDI and biological aging.

Biological aging	Model 1 [β (95% CI)]	*p*-value	Model 2 [β (95% CI)]	*p*-value	Model 3 [β (95% CI)]	*p*-value
Phenotypic Age (years)	−0.30 (−0.40, −0.21)	<0.001	−0.29 (−0.40, −0.20)	<0.001	−0.20 (−0.31, −0.10)	<0.001
Q1	1 (ref.)		1 (ref.)		1 (ref.)	
Q2	−0.44 (−0.81, −0.06)	0.024	−0.41 (−0.80, −0.01)	0.044	−0.11 (−0.59, 0.38)	0.700
Q3	−0.79 (−1.20, −0.41)	<0.001	−0.84 (−1.20, −0.43)	<0.001	−0.61 (−1.10, −0.10)	0.019
Q4	−1.20 (−1.60, −0.83)	<0.001	−1.20 (−1.60, −0.81)	<0.001	−0.82 (−1.30, −0.37)	<0.001
*P* for trend	<0.001		<0.001		<0.001	
Biological Age (years)	−0.76 (−0.91, −0.61)	<0.001	−0.70 (−0.85, −0.56)	<0.001	−0.54 (−0.69, −0.38)	<0.001
Q1	1 (ref.)		1 (ref.)		1 (ref.)	
Q2	−0.90 (−1.50, −0.31)	0.003	−0.66 (−1.30, −0.07)	0.030	−0.51 (−1.10, 0.12)	0.110
Q3	−1.60 (−2.20, −1.00)	<0.001	−1.40 (−2.00, −0.83)	<0.001	−1.10 (−1.80, −0.42)	0.002
Q4	−2.80 (−3.40, −2.20)	<0.001	−2.50 (−3.10, −1.90)	<0.001	−1.80 (−2.50, −1.10)	<0.001
*P* for trend	<0.001		<0.001		<0.001	
Telomere length	0.02 (0.01, 0.03)	<0.001	0.02 (0.01, 0.04)	0.021	0.01 (−0.01, 0.04)	0.200
Q1	1 (ref.)		1 (ref.)		1 (ref.)	
Q2	0.01 (−0.05, 0.04)	0.800	−0.01 (−0.05, 0.03)	0.600	0.01 (−0.08, 0.08)	0.900
Q3	0.01 (−0.06, 0.08)	0.800	0.01 (−0.06, 0.08)	0.900	0.01 (−0.08, 0.09)	0.900
Q4	0.08 (0.01, 0.15)	0.021	0.07 (−0.01, 0.14)	0.085	0.06 (−0.02, 0.15)	0.120
*P* for trend	0.021		0.082		0.112	
Klotho (pg/mL)	7.9 (3.3, 13.0)	0.001	8.3 (3.7, 13.0)	<0.001	6.2 (1.0, 11.0)	0.020
Q1	1 (ref.)		1 (ref.)		1 (ref.)	
Q2	0.59 (−18.0, 19.0)	0.900	1.30 (−18.0, 20.0)	0.900	−1.40 (−21, 18)	0.900
Q3	20.0 (2.6, 37.0)	0.024	26.0 (7.5, 44.0)	0.006	19.0 (−0.70, 40)	0.058
Q4	37.0 (15.0, 58.0)	0.001	39.0 (18.0, 61.0)	<0.001	32.0 (9.0, 55.0)	0.007
*P* for trend	0.001		<0.001		0.008	

Model 1: no covariates were adjusted.

Model 2: age, gender, education level, marital, PIR, and race were adjusted.

Model 3: age, gender, education level, marital, PIR, race, smoking, drinking, hypertension, diabetes, and high cholesterol were adjusted.

PHDI, Planetary Health Diet Index; PIR, Ratio of family income to poverty; OR, odds ratio; CI, confidence interval.

*β was calculated as per 10 scores increase in PHDI.*

Apart from this, a noteworthy positive relationship between PHDI and klotho was revealed by the fully adjusted logistic analysis. As every 10 scores in PHDI increased, the klotho showed an elevation of 6.2 pg/mL [6.2 (1.0, 11.0)]. Furthermore, the result of the trend test approved the connection when PHDI was regarded as a categorical variable (*P* for trend = 0.008), with individuals in the Q4 group of PHDI having 30.2 pg/mL [30.2 (9.0, 55.0)] in klotho increase than those in the Q1 group. In Model 2, telomere length increased by 0.02 bp for every 10-point rise in PHDI, even after adjusting for age, gender, education level, marital, PIR, and race.

**Figure 2:**
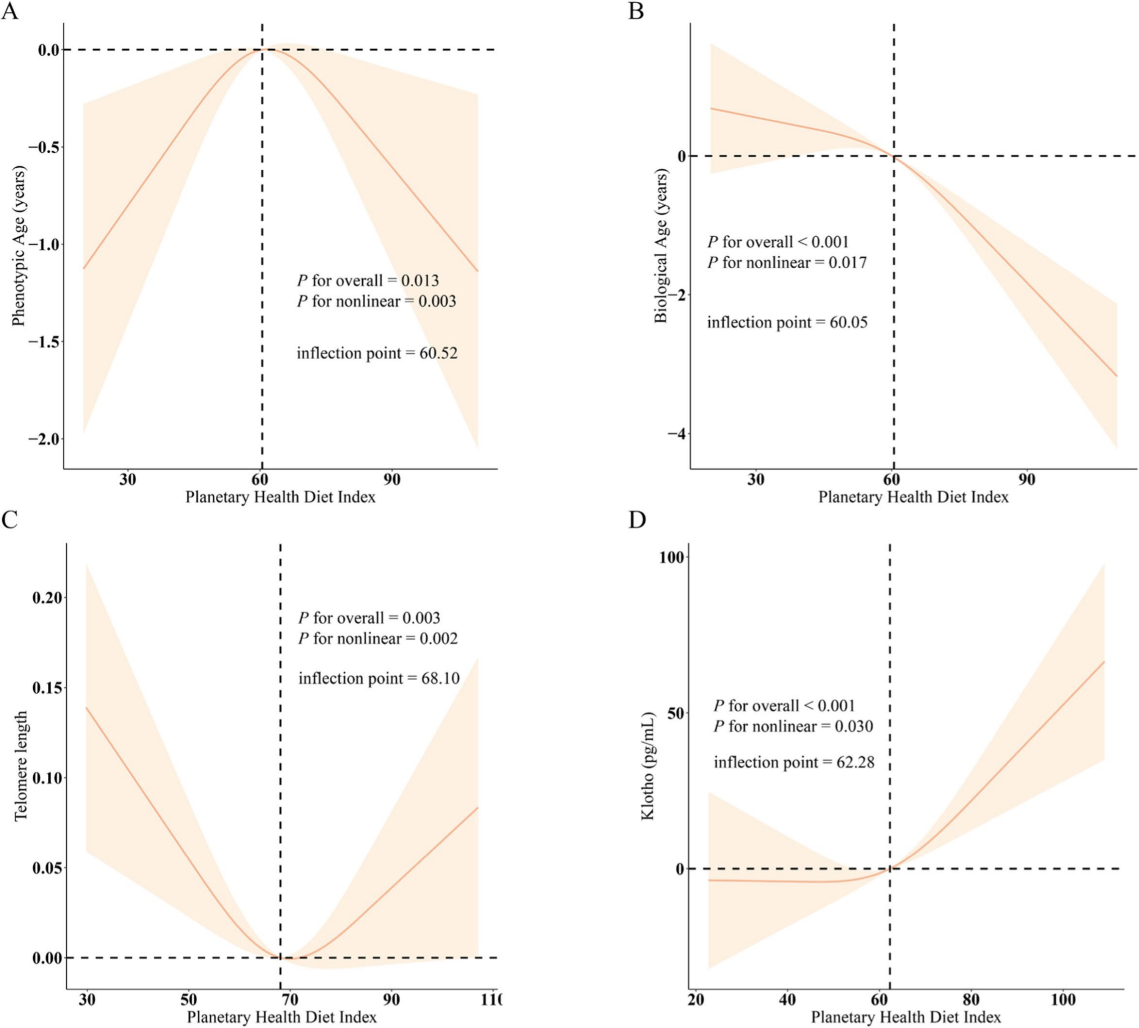
The nonlinear associations between PHDI and biological aging. (A) PHDI and Phenotypic Age; (B) PHDI and Biological Age; (C) PHDI and Telomere length; (D) PHDI and Klotho; *β* (solid lines) and 95% confidence levels (shaded areas) were adjusted for age, gender, education level, marital, PIR, race, smoking, drinking, hypertension, diabetes, and high cholesterol.

**Figure 3:**
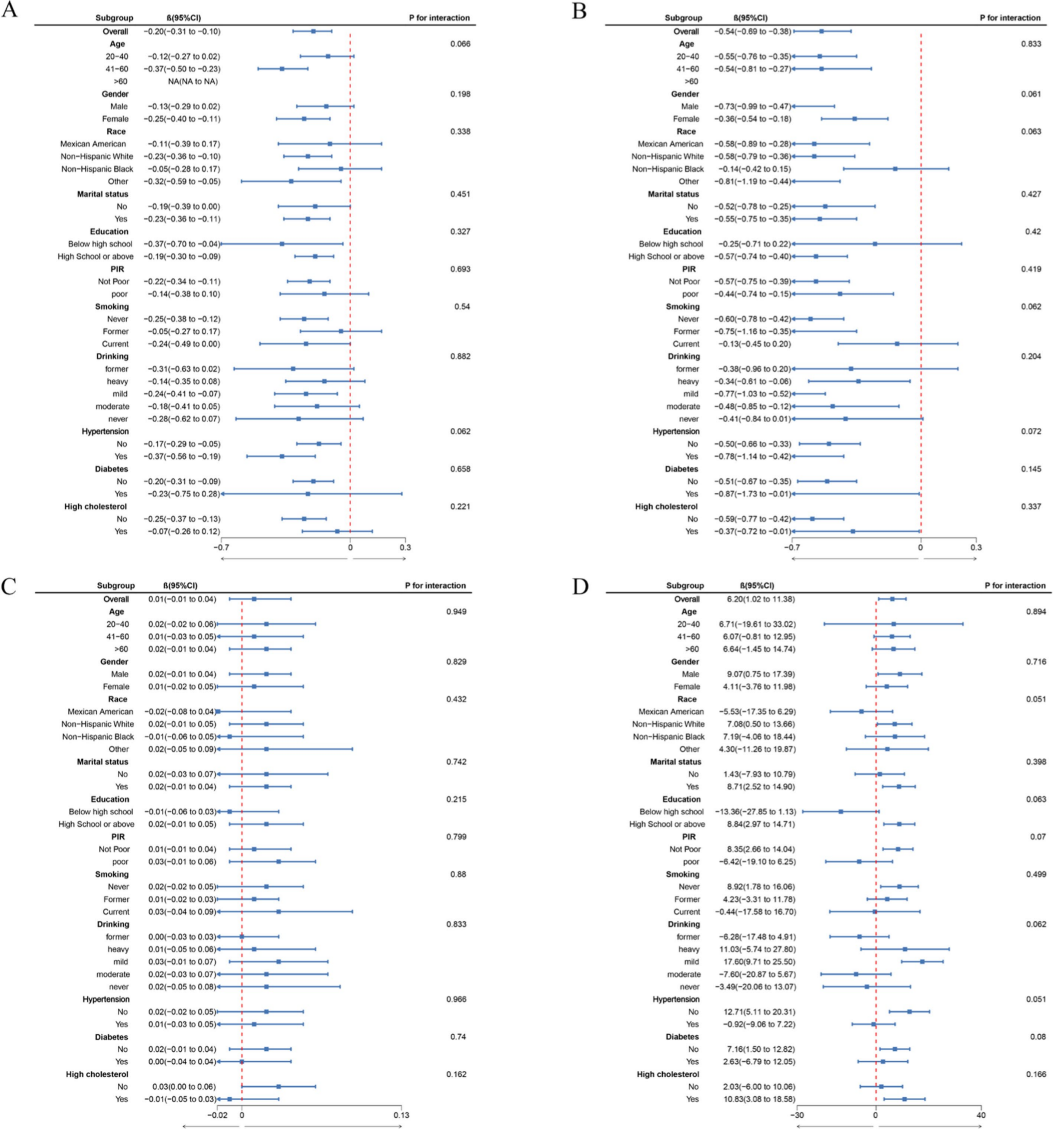
Subgroup analysis between PHDI and biological aging. (A) PHDI and Phenotypic Age; (B) PHDI and Biological Age; (C) PHDI and Telomere length; (D) PHDI and Klotho; *β* was calculated as per 10 scores increase in PHDI. Analyses were adjusted for age, gender, education level, marital, PIR, race, smoking, drinking, hypertension, diabetes, and high cholesterol.

Additionally, the RCS analysis revealed the association between PHDI and four indicators from significant curvilinear prospectives according to Figure 2. The inflection points for phenotypic age, biological age, telomere length, and klotho were 60.52, 60.05, 68.10, and 62.28, respectively. Besides, we found no noteworthy interactions in subgroup analysis, which evaluated potential variables influencing the connection between PHDI and four indicators (Figure 3).

## 4 Discussion

In this large-sample population-based study, we disentangled that high adherence to the PHD, as quantified by PHDI, had a significant relationship with delayed biological aging. To validate our findings, we measured biological aging as four renowned indicators, of which higher PHDI was inversely associated with phenotypic age and biological age. In contrast, higher PHDI showed a positive connection with higher klotho levels.

Until this point, there are no certified pharmacological methods for healthy aging. Thus, dietary interventions become the cornerstone. Multiple diets have gained popularity in slowing down biological aging and have been extensively investigated in previous research. For instance, the most popular vegetable-rich dietary model, the MedDiet, had the anti-aging ability to decelerate the shortening of telomeres based on diverse epidemiological evidence (39). Adherence to the MedDiet was also significantly associated with negative PhenoAge advancement and higher soluble klotho levels, according to Thomas A et al. study and Wu SE et al. study (40, 41). As for other dietary patterns, Kawamura T et al. reported that healthy Japanese dietary pattern was inversely correlated with two kinds of epigenetic age accelerations, including FitAgeAccel and AgeAccelGrim (42). Kim Y et al. calculated scores for the Dietary Approaches to Stop Hypertension (DASH) diet and found a protective effect of the DASH diet on epigenetic aging (43). Taken together, prior epidemiological studies showed that present dietary patterns seem promising for managing biological aging. However, these studies primarily relied on a singular assessment method, which may not fully capture the complexity of the biological aging process. In contrast, we employed four multifaceted indicators associated with biological aging, providing more comprehensive effects of PHD on decelerating biological aging, ultimately contributing to a deeper insight into its potential benefits for human longevity.

The impact of the Planetary Health Diet (PHD) on overall life expectancy is a significant topic of discussion, with related studies exploring the relationship between PHD or associated dietary health and biological aging. For instance, a prospective cohort study by Chen et al. (25) investigated the effects of plant-based and planetary health diets on mortality risk among middle-aged and older populations. Similarly, Hu (26) examined dietary strategies for promoting healthy aging and longevity from an epidemiological perspective, underscoring the importance of the relationship between PHD and biological aging. Furthermore, research by Wu et al. (28) and Shen et al. (27) indicated that adherence to the “Eight Basic Principles of Better Living” is closely linked to the biological aging process, as well as the association between plant-based diets and the gut microbiome.

Unlike conventional dietary patterns prioritizing nutritional adequacy and overlooked environmental concerns, the PHD is the first diet designed to meet the challenges of feeding a growing population while preserving our planet’s health. Otherwise, if we keep on ignoring the detrimental impacts of traditional industrial food systems on exacerbating food insecurity and natural resource depletion, 30% of the world population will suffer from hunger by 2050 (44). In terms of planetary health, the PHD supports sustainable management of agricultural resources by minimizing processed food and animal-sourced productions, while confronting environmental problems including soil degradation, deforestation, nitrogen and phosphorus pollution, water scarcity, and greenhouse gas emission (32, 45, 46). Besides, the PHD provides numerous benefits by ensuring the intake of micronutrient-rich foods containing essential vitamins, minerals, and phytonutrients, which are crucial for maintaining dietary health. Generally speaking, adopting the PHD could serve as a vital step toward environmental sustainability and human well-being, making it a superior choice for those seeking to impact global challenges positively.

The underlying mechanisms that drive the negative association between PHD and biological aging are unknown, but several potential hypotheses can be proposed. The antioxidant and anti-inflammatory properties of PHD may highlight its importance in aging deceleration. Firstly, immunosenescence is a critical consequence of oxidative stress in the aging process, with neutrophil dysfunctions of deteriorated phagocytic capability, abnormal adhesion, and chemotaxis (47). Vitamin C and vitamin E, abundant nutrients in the PHD serving as powerful antioxidants, can improve the immune function of neutrophils (48). Antioxidants reduce oxidative stress through multiple mechanisms, thereby influencing cellular senescence. Firstly, they directly scavenge excess free radicals in the body, thereby reducing damage to cell membranes, proteins, and DNA. Secondly, these substances can upregulate the expression of intracellular antioxidant enzymes, such as superoxide dismutase and glutathione peroxidase, enhancing the cell’s antioxidant capacity. Additionally, they protect mitochondrial function, reducing the production of reactive oxygen species (ROS) and subsequently lowering oxidative stress. Moreover, adherence to the PHD for at least 1 month can reshape the human microbiome structure and induce growth in *Bifidobacterium adolescentis* (*B. adolescentis*) (49). The contribution of *B. adolescentis* to extending lifespan and health span was confirmed by Chen S et al. study. They discovered that in Terc−/− animal models, *B. adolescentis* was involved in the upregulation of a crucial reactive oxygen species (ROS) scavenger named catalase (CAT) enzyme, and altering oxidative stress-associated metabolites (50). Another additional but important consideration is how PHD may help prevent biological aging by modulating cellular autophagy. The clearance and degradation of excessive or harmful components through autophagy are the keys to maintaining cellular metabolic balance and are closely associated with aging progress (51). The PHD has a rich dietary fiber source, which is a major substrate for short-chain fatty acids (SCFAs). Previous research has demonstrated that SCFAs regulated the process of autophagy mainly through AMPK/mTOR and PI3K/AKT/m TOR signaling pathways in aging-related diseases (52, 53). In addition to other micronutrients in PHD, several epidemiological studies revealed that dietary folate and carotenoid intake may also play essential roles in increasing klotho levels (54, 55). Given these limited findings, the lack of mechanical research leaves gaps in understanding how specific nutrients modulate klotho levels. More controlled experimental studies are needed to address these problems.

Our study gave the first new insight into the association between adherence to PHD and biological aging, which assessed a large sample of more than 40,000 individuals over 15 years. The reliability of our study was enhanced by utilizing several indicators, providing a more extensive understanding of how PHD promotes longevity across different physiological domains. These findings not only broaden the PHD clinical significance but also provide valuable guidance on dietary strategy for slowing down biological aging, with advantages that extend from planetary health to dietary health. Meanwhile, some limitations should also be acknowledged. Firstly, our study cases did not encompass other ethnic populations except Americans, which may make the results not generalizable and replicable enough. Secondly, we failed to investigate the causality between PHD and biological aging due to conducting a cross-sectional study. Thirdly, food consumption information was gathered through 24-h diet recall questionnaires at a single time, raising the possibility of response bias and not representative of long-term alterations in food intakes. Lastly, other potential residual confounders cannot be wholly figured out despite adjusting for many relevant covariates.

## 5 Conclusion

In conclusion, our study explored a significant correlation between PHDI and biological aging, indicating that adherence to PHD may be closely linked to reducing biological aging. It is promising that adopting PHD could profoundly impact both longevity and health span. Additional studies are needed to confirm the potential anti-aging effects of PHD and to understand underlying mechanisms better.

## Data Availability

The datasets presented in this study can be found in online repositories. The names of the repository/repositories and accession number(s) can be found at: Publicly available datasets were analyzed in this study. The data can be found at: https://www.cdc.gov/nchs/nhanes/.

## Footnotes

^1^The study’s webpage is located at https://www.cdc.gov/nchs/nhanes/irba98.htm
