# Supplementary material for: Association between the Planetary Health Diet Index and biological aging among the U.S. population
Source: Front Public Health. 2024 Oct 22;12:1482959. doi: 10.3389/fpubh.2024.1482959 (PMC11534585; doi:10.3389/fpubh.2024.1482959)
Supplement: Supplementary file 1 [file Table_1.DOCX]

**Supplementary Material**

| **Formulae for biological age and phenotypic age.**   1. Phenotypic age was determined through the application of the subsequent formula, in which, $\text{Phenotypic age }=141.50+\frac{Ln\left[-0.00553\times L\text{n}\left(\text{exp}\left(\frac{\left(\text{-1.51714}\times\text{exp}\left(\text{xb}\right)\right)}{0.0076927}\right)\right)\right]}{0.09165}$    xb=−19.907−0.0336×Albumin+0.0095×Creatinine+0.1953×Glucose+0.0954×LnCRP−0.0120×Lymphocyte Percentage+0.0268×Mean Cell Volume + 0.00188×Alkaline Phosphatase+0.0554×Leukocyte Count+0.0804×chronological age.   1. Klemera introduced a method for determining biological age using a set of eight biomarkers (Ln-CRP, serum creatinine, glycated hemoglobin, serum albumin, serum total cholesterol, serum urea nitrogen, serum alkaline phosphatase, and systolic blood pressure). The variables j and i represent the number of biomarkers and samples, respectively. The parameters k, q, and s denote the slope, intercept, and root mean square error of the regression analysis between biomarkers and chronological age. The variance explained by the regression of biomarkers against chronological age is plotted as r_j_^2^.     **Formulae for telomere length.**  Quantitative PCR methods were then employed to measure telomere length (T/S ratio) relative to standard reference deoxyribonucleic acid (DNA). 20 The T/S ratio, which refers to the Telomere‐to‐Single copy gene ratio, was further converted to base pairs (bp) using the formula: base pairs = 3274 + 2413 × [T/S], calculated by comparing the telomere restriction fragment (TRF) length analyzed via Southern blot and the T/S ratio of DNA samples using human diploid fibroblast IMR90.  **Table S1.** Scoring criteria for the Planetary Health Diet Index (PHDI). | | |
| --- | --- | --- |
| **Dietary component** | **Category minimum score** (0 points) | **Category maximum score** (10 points) |
| ***Adequacy components*** | | |
| Whole grains^1^ | 0 grams | ≥ 75 grams for women  ≥ 90 grams for men |
| Whole fruits (excludes fruit juice) | 0 grams | ≥ 200 grams |
| Non-starchy vegetables | 0 grams | ≥ 300 grams |
| Nuts and seeds | 0 grams | ≥ 50 grams |
| Legumes |  |  |
| Non-soy legumes^2,3^ | 0 grams | 100 grams |
| Soybean/ soy foods^2,3^ | 0 grams | 50 grams |
| Unsaturated oils | 0% of total energy intake | ≥ 10% of total energy intake |
| ***Moderation components*** | | |
| Starchy vegetables | ≥ 200 grams | ≤ 50 grams |
| Dairy^4^ | ≥ 4.08 cup-equivalents | ≤ 1.02 cup-equivalents |
| Red and processed meat | ≥ 300 grams | ≤ 14 grams |
| Poultry | ≥ 58 grams | ≤ 29 grams |
| Eggs | ≥ 120 grams | ≤ 12 grams |
| Fish | ≥ 50 grams | ≤ 15 grams |
| Saturated oils and *trans* fats | ≥ 21% of total energy intake | ≤ 3.5% of total energy intake |
| Added sugar and fruit juice | ≥ 25% of total energy intake | ≤ 5% of total energy intake |
| ^1^Thresholds were based on the midpoint of the recommended range listed in EAT-Lancet Commission Scientific Report.  ^2^Grams per day calculated from dry weight.  ^3^To calculate the score for the legumes component, the nonsoy and soy subcomponents were each weighted at 0.5.  ^4^In FPED, 1 serving of dairy is equal to 245 g of whole-milk or derivative equivalent. In the EAT-Lancet report, scores were assigned ≤250 g whole-milk or derivative equivalent for the maximum score or ≥1000 g whole-milk or derivative equivalent for the minimum score.  Note: The above dietary components were obtained from the Food Pattern Equivalence Database (FPED) of the 24-hour dietary recall data(https://www.ars.usda.gov/northeast-area/beltsville-md-bhnrc/beltsville-human-nutrition-research-center/food-surveys-research-group/docs/fped-databases/). | | |

**Table S2.** Description of covariates

| Covariates | Description in NHANES |
| --- | --- |
| Age | Divided into three groups: 20-40 years old, 41-60  years old, >60 years old |
| Gender | Male and Female |
| Race | Mexican American, Non-Hispanic Black, Non-Hispanic White, Other Race |
| Educational level | Below high school, High School or above |
| Marital status | Yes: Married/Living with partner |
| PIR | Poor: <1.3; Not Poor:>=1.3 |
| Obesity | Yes: BMI>=30 |
| Smoking | Smoking status was grouped into never smoker (defined as <100 cigarettes in a lifetime), current smoker (defined as ≥100 cigarettes in a lifetime), and former smoker (defined as ≥100 cigarettes and had quit smoking) |
| Drinking | heavy drinking (≥4 drinks/day for men, ≥3 drinks/day for women, or ≥5 days of drinking in a month),  moderate drinking (≥3 drinks/day for men, ≥2 drinks/day for women, or ≥2 days of drinking in a month),  mild drinking (≤2 drinks/day for men, ≤1 drink/day for women, and ≥12 drinks in a year),  and never-drinking (total number of drinks in a year <12, and dietary alcohol content of 0%) |
| Physical activity | Active physical activity was defined as >599 MET, or >149 min of moderate physical activity, or >74 min of vigorous physical activity |
| Diabetes | Diabetes was defined as a history of previous diabetes, HbA1c level ≥6.5%, or fasting blood glucose level ≥126 mg/dL |
| Hypertension | The diagnostic criteria consist of self-reported hypertension history, the utilization of antihypertensive medication, a systolic blood pressure (SBP) ≥ 140mmHg, or a diastolic blood pressure (DBP) ≥ 90mmHg |
| High cholesterol | Participants were asked whether they had high cholesterol |

PIR, Ratio of family income to poverty
